# Use of Video Consultations for Patients With Hematological Diseases From a Patient Perspective: Qualitative Study

**DOI:** 10.2196/11089

**Published:** 2018-12-19

**Authors:** Nina Primholdt Christensen, Dorthe Boe Danbjørg

**Affiliations:** 1 Odense Patient Data Explorative Network Hematological Research Unit Odense University Hospital Odense Denmark; 2 Centre for Innovative Medical Technology Department of Hematology, Odense University Hospital University of Southern Denmark Odense Denmark

**Keywords:** consultations, telehealth, technology, patient, hematology

## Abstract

**Background:**

The need for the use of telemedicine is expected to increase in the coming years. There is, furthermore, a lack of evidence about the use of video consultations for hematological patients, and how the use of video consultations is experienced from the patients’ perspective.

**Objective:**

This study aimed to identify patients’ experiences with the use of video consultations in place of face-to-face consultations, what it means to the patient to save the travel time, and how the roles between patients and health care professionals are experienced when using video consultation. This study concerns stable, not acutely ill, patients with hematological disease.

**Methods:**

The study was designed as an exploratory and qualitative study. Data were collected through participant observations and semistructured interviews and analyzed in a postphenomenological framework.

**Results:**

The data analysis revealed three categories: “Intimacy is not about physical presence,” “Handling technology,” and “Technology increases the freedom that the patients desire.”

**Conclusions:**

This study demonstrates what is important for patients with regards to telemedicine and how they felt about seeing health care professionals through a screen. It was found that intimacy can be mediated through a screen and physical presence is not as important to the patient as other things. The study further pointed out how patients valued being involved in the planning of their treatment. The patients also valued the freedom associated with telemedicine and actively took responsibility for their own course of treatment. Patients felt that video consultations allowed them to be free and active, despite their illness.

## Introduction

### Background

Hematological patients in Denmark may spend several hours traveling to the hospital as the treatment is centralized at a few university hospitals [1]. Therefore, the purpose of this study was to examine how hematological patients experience the use of telemedicine. Telemedicine is defined by the World Health Organization as “the delivery of health-care services, where distance is a critical factor” [2]. Thus, the concept refers to a range of different services, for example, monitoring, treatment, and communication based on different telemedicine technologies. This project will focus on video consultations instead of or as a supplement to a physical consultation.

Several pilot projects covering telemedicine have been conducted in Denmark. These projects have shown that telemedicine provides benefits in the shape of more self-supporting patients, economical benefits, and contiguous patient processes [3]. In a Danish study from 2016, telemedicine was investigated in patients with chronic obstructive pulmonary disease. It was found that teleconsultation was experienced as qualified and facilitated a close relation between the nurse and the patient. Furthermore, the patients became more active participants in their own treatment because they had to be more active in relation to the technical aspects and responsibility toward their own disease [4].

In general, it has been emphasized that telemedicine holds great potential for the delivery of health care services by enhancing access, quality, efficiency, and cost-effectiveness. It is predicted that telemedicine will continue in the relocation of health care delivery from the hospital or clinic into the home [5]. However, it is also emphasized that policy makers should be cautious about recommending increased use of, and investment in, unevaluated technologies [6]. Hailey et al [7] stated that research within telemedicine has been inadequate, and there is a need for investigations into the newer telemedicine solutions for well-defined patient groups.

Yet no projects involving patients with hematological diseases have been conducted in a Danish context, and a systematic database search shows very limited research on video consultations with patients with hematological diseases globally. The majority of the studies were derived from Australia and the United States, and only few European studies appeared in the search. In most of the studies found concerning telemedicine and hematology, telemedicine is used because of the long distances and to keep the specialized doctors available for patients despite the long distances. In a study from Kansas [8], the perceptions of telemedicine of 22 patients with hematological diseases were explored. In this study, the majority of patients expressed satisfaction with replacing the face-to-face contact with a video consultation, allowing them to consult a specialist. The study showed that the patient and the doctor have different roles according to and during the consultation but did not explore how this is evident. Another aspect is the audio-visual differences caused by the screen that make the consultation different from a face-to-face consultation. The study also indicated that some aspects of the communication were found to be inhibited because of the absence of personal contact and the insecurity about the technology. The study showed that further research is needed to explore how patients with hematological diseases experience their role during a video consultation and which communicational barriers they might experience.

Dinesen et al focus on the importance of defining which group of patients are suitable for using telemedicine and conclude that there is no *one-size-fits-all* approach when it comes to the use of telemedicine [9]. The diagnoses and patients differ, and there is a need for further investigation of the combination of technology and different patient groups. Kidholm et al [10] furthermore define that it is important to find the group of patients who can benefit from the use of telemedicine before the benefits show. They also define that telemedicine cannot stand alone without defined groups of patients [10].

In the future, the intention for the hospitals is to have fewer admitted patients and instead treat the patients in their own homes [11]. The need for use of telemedicine is expected to increase during the coming years, and therefore, it becomes important to gain knowledge about the use of video consultation for patients with hematological diseases as seen from the patients’ perspective.

### Aim

The overall aim was to explore how patients experience the use of video consultations. This will be uncovered through the following research questions:

- How do patients experience the use of video consultations in place of a face-to-face consultation?
- How do they experience the lack of physical contact?
- What does it mean to the patient to save the travel time?
- How are the roles between patients and health care professionals experienced when using video consultation?

## Methods

### Design

The study was designed as an exploratory and qualitative study.

### The Intervention

This research project is part of a larger pilot study, where patients with hematological diseases from a small island to the south of Funen were given the opportunity to talk to a hematologist from the outpatient clinic in Odense through a video screen, while the patient is located at the local hospital on the island instead of a face-to-face consultation.

The intervention was initiated in cooperation between the municipality on the island, the Innovation Department at Odense University Hospital, and the Hematological Research Unit. In addition, 2 identical video screens were bought for use at the hospital on the island, and 1 screen was placed in the outpatient clinic in Odense.

The pilot study was initiated in April 2017 and continued until the end of December 2017. A total of 17 patients with different diagnoses have been included in the pilot study. The video consultations were used both for monitoring and treatment of the patients. Some patients got their blood pressure measured by the nurse at the local hospital. The doctor used the video consultation in combination with the blood results to determine whether the patients were suitable for next treatment or a new kind of treatment.

A research nurse from the Hematological Research Unit informed the patients about participation in the video consultations and initiated the video consultation with the hematology specialist. On the island, the patients were offered to have the nurse to participate, if the patient expressed a need for this. All patients were helped by a nurse to enter the room, and the nurse also provided assistance with the technology.

#### Sample

**Figure 1:**
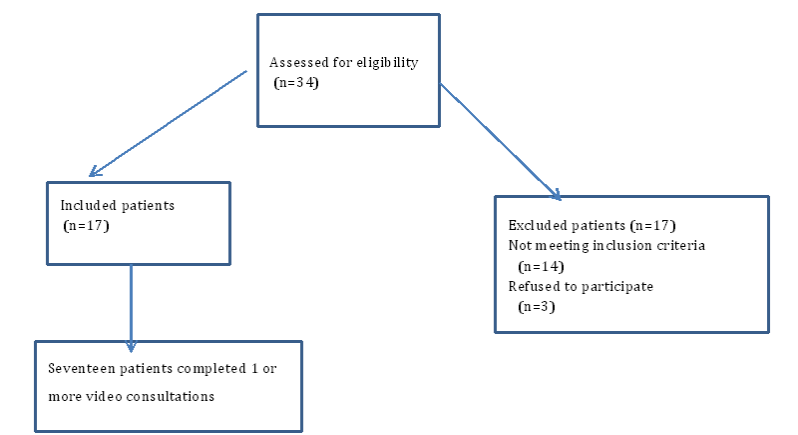
The Consolidated Standards of Reporting Trials (CONSORT) diagram.

The study population is patients with hematological diseases living on the small island south of Funen (see Figure 1 for the selection process).

#### Inclusion Criteria

Inclusion criteria include patients with hematological diseases who can come to the hospital on the small island and who have participated in a video consultation in the pilot study, patients who can be assessed through video consultation, and patients who have been approved to participate in the video consultation. The approval was made by a hematology specialist who last saw the patient in the outpatient clinic. The hematology specialist selected patients who were in a period with stable disease and patients who did not receive intravenous treatment. Patients with all kinds of hematological diseases could be selected. After being evaluated for inclusion, the patient signed the informed consent form.

#### Exclusion Criteria

Exclusion criteria included patients who have not been approved by the hematology specialist to participate in a video consultation because of their specific diagnosis or general condition, for instance, if they were in an unstable period in their disease.

### Data Collection

#### Participant Observation and Semistructured Interviews

Participant observations [12] of the patients during video consultations were conducted from November 2017 to December 2017. The first author made the observations. The observations were based on an observation guide that structured the focus as well as the field notes that were written during the observations. The observations provided an opportunity for open and informal follow-up interviews with patients regarding their immediate experiences with the video consultations. Furthermore, semistructured interviews with patients were conducted at the patients’ homes on the small island.

A semistructured interview guide [13,14] was compiled focusing on the following themes: (1) technology issues; (2) how the consultation is experienced when it is technology mediated; (3) communication through a screen; (4) roles (health care professionals and patients and relatives [if applicable]);(5) everyday life and living with a hematological disease, and experiences of being ill; and (6) what impact does the long travel time to Odense have on the patient, and what difference the video consultation has made for the patient. The interviews lasted 30 to 50 min and were audio-recorded and transcribed verbatim. During the interviews, the patients were asked what their information technology (IT) skills were and if they used a computer, tablet, or mobile phone in their everyday lives.

#### Analysis

The collected data were analyzed in a postphenomenological framework with focus on technology-mediated perception, transformation, and constitution [15-18]. To organize the analysis process, we followed the steps from “systematic text condensation” [19-21]. The analysis was organized according to the steps taken in the analysis, as shown in Table 1.

**Table 1:** The analysis process—examples from the analysis.

Themes^a^	Codes^b^		Meaning^c^
	Quotation	Code	
Demanding travel time	“It is such a hard trip. It takes all day—I’m picked up at appr. 6:45 or thereabouts, and I am back home at 4-4:30 pm and I also need to cook dinner and stuff like that. Yes, and then I fall asleep and sleep right through to the next day.”	Everyday life	Freedom
Eye contact	“Yes...yes, we did—really good contact. I looked at him, and he looked at me, and I actually rather liked that he was over there.” (laughs)	Intimacy	Intimacy

^a^Refers to superior themes extracted after the first open reading (step 1).

^b^Refers to transition from themes to codes and identifying meaningful units (step 2). The meaningful units are coded based on the superior themes.

^c^Refers to transition from codes to meaning (step 3). The meaningful units are sorted into groups.

First, we captured an overall impression of the data, thereby obtaining a preliminary set of main themes. Second, the data were separated into meaningful topics relevant for the research question. Third, the meaningful topics were coded, and the topics were condensed. Finally, the findings were synthesized, involving a shift from condensation to descriptions and categories. The codes were developed based on the preliminary themes identified in the first step and the theoretical framework.

To enhance validation, the first and second authors worked on the analysis together. They discussed their overall impression of the data and then they highlighted the meaningful topics with a marker, and the codes were discussed between the 2 authors. The first author wrote down the analysis. The findings were then discussed in relation to relevant literature and a postphenomenological framework with focus on technology-mediated perception, transformation, and constitution.

#### Ethics

During data collection, the researchers were continuously reflective about the aim of the study and the methods applied [22]. Furthermore, the participants were informed thoroughly about the project, so they could make an informed choice on whether to enroll in the study or not. The patients were granted anonymity.

The study was registered with the Danish Data Protection Agency (2012-58-0018). The data are stored in a secure SharePoint site.

## Results

### Overview

The study included 12 participants aged 55 to 86 years with different hematological diagnoses. All participants were in a stable period in relation to their disease during the time when the video consultations took place (see Multimedia Appendix 1 for details about age, gender, diagnosis, occupation, and educational level).

The majority of the interviews took place in the participants’ own homes—only 1 interview took place at a participant’s workplace. Moreover, 5 participants were observed during a video consultation at the local hospital. Results from the interviews and the participant observations revealed 3 categories reflecting the participants’ experiences with video consultations. These categories are “Intimacy is not about physical presence,” “Handling technology,” and “Technology increases the freedom that the patients desire.” These themes will be described in more detail below.

### Intimacy is not About Physical Presence

The analysis of the interviews revealed that participants found different things important in a consultation with the doctor. The participants found that they had a closer and more focused consultation with the doctor during the video consultation than during a consultation with physical presence. The reason was that the doctor was looking at and talking more directly to them—they felt intimacy and also a higher degree of eye contact, which was important to most of the participants:

No, I feel that we have eye contact (the dog barks). Even if it is through a screen, I feel that we have eye contact.

Male 4, 77 years

A woman on the labor market stated the following, which accurately describes the intimacy and difference between a normal consultation and a video consultation:

Yes, there was only me and no noise, and he had to look at me and less at the computer (laughs), and we had a dialogue, and he couldn’t just walk away from the screen, could he (laughs), or anything else, and now there’s focus, and the contact was really good, so I would like to do it again.

Female 11, 60 years

During the observations, it was noted that the participants used their body language a lot; they were looking directly at the screen and were very active with their body movements, yet at the same time they appeared relaxed. None of the observations showed that the participants were acting insecure or uncomfortable with sitting in front of the video screen. When the participants were asked if they missed the physical handshake, one of the participants expressed the following:

Interviewer: You were not unsure because you couldn’t give a physical handshake?

Woman: Not at all – no, I don’t think so. There are little words you can use instead, “hugs” and “hello,” and things like that.

Female 6, 55 years

As the above quote indicates, the verbal and nonverbal communication changes when the consultation is conducted as a video consultation. It was also observed that the participants often waved to the doctor when they said “hi” and “goodbye,” and they were laughing and smiling a lot.

The interviews and observations showed that the interior decoration of the rooms where the video consultation was performed did not play an important role in the participants’ experience of the video consultation:

Interviewer: It doesn’t sound like the room scared you off?

Male: No, no, not at all.

Interviewer: It is after all a rather special room – something like an operating room.

Male: No, that didn’t matter – I knew that I wasn’t going on the operating table and under the knife (Laughs).

Male 8, 70 years

Most of the participants mentioned that that the doctors examined them quite infrequently during a consultation with physical presence and that the content of the consultation mostly was a talk about the blood results and side effects—a talk that they experienced during this project could be replaced by a video consultation. The participants for this study were all stable in their disease. When they were asked whether they preferred to sit in front of the doctor physically or via a video screen if they were to receive bad news, most of the participants preferred to sit in front of the doctor physically. However, some participants preferred the opposite, as is reflected by what 1 male participant said:

Yes, and I would say that actually – theoretically that if I were to receive very serious news – if you think about that scenario – then I can see that you would react right off the bat and think that no, I’ll come over and hear it directly from you. But on the other hand, you could say that it would be less stressful just sitting by the video screen there and talk nice and quietly with the people in Odense who you would talk to anyway and then not have to go on a difficult journey afterwards and then be at home, but instead you can make it home in 5 minutes, and my wife could be involved in the consultation on the screen, if she wanted to – however, we haven’t tried that yet. But she would, of course, in a situation like that, sit and act the same was as if it was in Odense, right.

Male 7, 69 years

It was essential to most of the patients that they could talk to their usual doctor during the video consultations.

To have met the doctor in real life before having a virtual consultation means that it felt more natural to switch to nonphysical consultation because the contact was already established in the physical room. It was furthermore very important for some of the participants that their doctor showed their knowledge about their disease:

I feel safe that my doctor knows my disease and knows what is going to happen and if I am to come in.

Female 9, 78 years

The results showed that intimacy in a consultation can manifest itself in many ways and that the verbal and nonverbal communication—whether it is present in the virtual or physical consultation—are of great importance to the patients’ experience of intimacy.

### Handling Technology

All participants express that both the sound and the image during video consultations have been of very good quality and that it is important for the overall experience of a video consultation. The fact that the participants have not encountered any technical issues was important for the overall experience and for the participants to feel safe:

Interviewer: And what about the quality of the picture and sound?

Male: It was outstanding – no problems at all. There really isn’t.

Male 4, 77 years

One patient also experienced that it was possible for the doctor to *examine* him through the screen:

We talked really well, and I was actually impressed that she saw that I had kind of a red spot up here (points to his forehead) and I stuck my head really close to the screen, and I think she could zoom or something, but that’s amazing. Therefore the quality is really good – and I got some cream I could apply.

A majority of all participants have expressed that they were familiar with the use of IT, and most of them either had their own computer, tablet, or mobile phone available (see Multimedia Appendix 1). Despite this, the participants expressed confidence in the fact that the video consultation was taking place at the hospital, where the staff was responsible for handling the technology and could assist in case of technical problems. The fact that the staff took charge of the technical aspects of the video consultation meant that the participants could concentrate on the actual conversation with the doctor. During some of the video consultations, the staff was placed in the same room in case of questions and as security.

During the patient observations, it was clear that the nurses who handled the screens and informed the patients had an important role—the patients asked them questions and followed their instructions:

Female: Yes, I sad that it was OK that she stayed in the room with me.

Interviewer: Yes, maybe it was a comfort for you, in case something happened.

Female: Yes, with the screen or something.

Female 2, 82 years

To the question of whether the patients were interested in the ability to speak with the doctor in their own home, the majority did not prefer this option. The reason was not a lack of IT skills but rather the feeling of too much responsibility. It was the fact of being responsible if the technology failed and therefore, also a risk of missing out on the time with the doctor:

In a way it is nice that the responsibility for the technology – someone over there has the responsibility for that – someone makes sure it works.

Male 7, 69 years

### Technology Increases the Freedom That the Patients Desire

The participants experienced that they saved themselves 6 to 8 hours of transportation with the ferry, car, or public transport and waiting time when they replaced the consultation in the outpatient clinic in Odense with the video consultation at the local hospital at the island. Saving traveling time had a great impact on all the participants’ feeling of freedom. Some participants felt weak, and the long travel time meant that they had to sleep and recover 2 days after a visit in Odense—the travel time was demanding and draining for especially the elderly and physically weak participants:

And now I hope that this telefunction gets up and running so I can save the trip to Odense – it means so much – then I don’t have to sleep 1-2 days after.

Female 6, 55 years

In addition, the more fit participants experienced benefits from the less time spent on travelling—it gave them time to spend on their interests and also the feeling of self-determination. The participants got the opportunity to decide what suited them best in the actual situation they were in. The participants expressed in different ways that they were longing for the daily lives they had before they got sick and the opportunity to save a whole day of transportation:

It frees up some resources in a different way – I get a day where I can do some things. The entire day is not taken up by having to go to Odense. I sometimes have several things I have to do in a day. Today I can spend the time on several things and I wouldn’t be able to do that if I was going to Odense. I am, among other things going out to play cards and I am having people over tonight – I wouldn’t be able to do that if I was going to Odense.

Female 5, 72 years

During the observations, the first author talked to a woman aged 72 years just before she was entering the video consultation. She looked a bit stressed and when the author asked for the reason, she said that she was so busy; because she found out that she has got extra time to spend as she did not have to travel to Odense, she had committed herself to baking pancakes for an old age home with 50 persons—she worked as a volunteer for 2 different organizations; these jobs meant a lot in terms of quality of life and made a daily life with relevant content for her.

One participant described how her everyday life was affected already the day before she was going to the outpatient day clinic:

Yes, the worst part is when we’re off to Odense and I have to go there – and you know what – I can’t hear anything. I saw the ear specialist the day before yesterday, and I have used a hearing aid for 16 years now. And when I have to get up in time in the morning, I can’t hear the alarm clock, even if I’ve set 2, and I have bought a special alarm clock to be placed under my pillow, but I’m afraid it doesn’t work. So when I’m going to Odense – and if I then wake up at 4 am, I am on pins and needles and get up and stay awake until 7am when I’m being picked up – otherwise I’m afraid that I will go back to sleep...

Female 2, 82 years

A female participant also described how she often got infected with a virus after being on the ferry together with a lot of people. The fact that she can look forward to less transportation was of enormous importance to her. The amount of saved travel time has a measurable and positive impact on the participants’ experience of freedom—especially when it is unclear to the patients why the doctors need to see them in person. On being asked when they found it necessary to travel to the hospital in Odense, an elderly woman aged 86 years and a woman on the labor market stated:

Only if I was to be examined and take my clothes off every time – but I’m not doing that at all – and then I can’t see why I should go in there.

Female 1, 86 years

But I have never heard that the doctor used that argument for me to come in, and I have never been examined when I’ve been in there. But it is a long time since I was in there as I always try to get out of having to go in and ask if they can’t just call me. As I say to them I can measure my blood pressure, and I have blood samples done here, and what else is there...I damn well know where my blood pressure is at. But that is my situation, and I know that situations are individual.

Female 11, 60 years

It was clear that the participants showed a great amount of responsibility in relation to their disease and that they were aware of what kind of information they needed. All of the participants knew their blood test results very well, and it was also the common talking point during the video consultations. Most of the participants showed that they were self-managing in the way they were dealing with their needs in relation to their disease, and it was shown across diagnoses, age, and sociodemographic background. One of the participants was still active on the labor market, and therefore the need for flexibility was high. She was also self-monitoring her blood pressure and paid close attention to her blood test results. However, she also emphasized that it was a mental strain being confronted with the memories from earlier admissions when she had to be at the hospital:

But when I’m kinda feeling good and everything is on track, I can’t see any reason for going in, and I quickly feel made to feel sick when I go in and see people with all that stuff we had coming out of our bodies – can you imagine that?

Female 11, 60 years

Throughout the analysis, it showed that all the participants had faith in the health system, the hospital, and the doctors they met during the treatment and control. The participants relied on the fact that they were treated and controlled correctly and also trusted that the doctors would contact them, if they needed to see them in exceptional situations:

But then I thought, oh, it’s going to be a long journey and long day, but if that’s the way she wants it that is what she is going to get. Of course she has to be allowed to say that I want to take a look at you. For she can see on our color and in our eyes if something is the matter with us and we can talk nice and quietly. But I feel safe about it – I feel really safe about it. And as I say – as long as nothing else is wrong with me, then that’s fine.

Female 3, 72 years

Most of the participants also lived near the local hospital and did not consider it difficult to go there. The desire to separate hospitals and homes was also one of the reasons why the participants expressed that they did not want to test video consultation from their own homes. Few of the participants stated that they would like to be placed in their own homes when talking to the doctor. They imagined that they would appreciate the flexibility with more time for their own interests. Participants with and without IT skills were interested in the possibility of video consultation from their own homes—so there was no relation between IT skills and the interest in trying the consultation in the participants’ own homes.

One of the participants had a summerhouse in the north of Jutland, and when he found out that it was possible to have a video consultation from his summerhouse instead of driving back to the hospital in Odense, he became very satisfied with the flexibility it gave him and his wife:

Interviewer: Do you have a good internet connection at your summer house?

Male: Yes, we do.

Interviewer: Then that might be a possibility.

Male: Yes, and take your iPad with you and hook it up. No, we wouldn’t go home for that...

Male 12, 74 years

The participants’ basic trust in the doctors had a great impact on the way the video consultation was used between the participants and doctors, and it also had an impact on their roles. During the observations, it was seen that the patients acted naturally and were asking the same number of questions—only the doctors were experiencing less distractions and according to the participants, the doctors seemed more focused in the communication.

## Discussion

### Principal Findings

The purpose of this study was to investigate how hematological patients experienced the use of video consultations in the outpatient clinic. Some of the main findings in this study were that the participants experienced a valuable feeling of freedom because they did not have to spend time traveling to see the doctor, and they could maintain their everyday lives. The participants valued that they were given the option not to go to the hospital in Odense, so they could spend time on what was important and not being reminded about the time they were admitted and sick. The study also showed that the participants experienced a higher level of intimacy and self-determination during the video consultation because it was experienced that the doctor was more focused during the consultation.

### The Patient Role

The patients appreciated that they were able to feel normal and not feel like a patient when they were in a stable period. It gave them a feeling of freedom. Studies from Australia and Denmark also showed that patients are very satisfied with the use of video consultation and that it brings out the patient to play a more active role in their own treatment and care [4,23].

The participants’ everyday lives were affected by the fact that they had to travel for many hours to get to the consultation in Odense, and it gave them a feeling of decreased freedom. This is supported by a study concerning outpatient management of acute leukemia patients [24]. This study found that it was of great importance that the patients could maintain a normal everyday life together with their families and also be physically active. Most of the patients valued the feeling of being independent in the form of spending their time as they wanted. They did not feel they were able to do that when they were at the hospital because of waiting time for procedures and for the doctors to show up. A normal everyday life was of greatest importance [24].

The patients in the study can be viewed as self-managing as they all take an interest in their blood counts, and they often *ran* the consultations with questions. The study by Olesen points out that the patients are not automatically being empowered just because they are self-managing because the health professionals can be viewed as *their employer* who gives the patients tasks to solve in relation to their disease [25]. Olesen speaks about the patient as the *unpaid employee* [25]. However, the participants in this study expressed that they were empowered by the use of the video consultations because of the possibilities and the freedom the video consultations gave them. They felt that being able to participate in video consultations means that the disease does not have to control their lives, and thereby, they regained some control over their own lives. An Australian study also shows that involving patients as partners in the delivery of health care can make the development of new telehealth care solutions easier [26].

All patients with hematological diseases are asked to be aware of specific symptoms when being discharged from the hospital. Therefore, it was not new for the patients in this study to be aware of symptoms; however, they were given a more specific task as the doctor could not examine them, and one of the participants self-monitored her blood pressure. To be self-monitoring as a patient means that the patients can learn to understand themselves in a new way, which means that they can see themselves as responsible patients who react to their own symptoms and decide what kind of consultation they need at the time in question [27].

This was valued by the participants in our study as it was the possibility to decide for themselves. The participants could decide (if they lived up to the inclusion criteria) if they wanted a face-to-face consultation at the hospital or a video consultation. This is in line with the guidelines from the National Board of Health in Denmark [28]. The purpose of the guidelines is to support the health professionals to involve the patients in their own treatment and follow-up with the goal to increase patient satisfaction. They were showing signs of being active patients and expressed satisfaction about the option provided for an alternative way to complete a consultation. It became clear that the patients desired to feel free, that going to the hospital for consultations and checkups was both time consuming and emotionally and physically draining, and they experienced that video consultations were less stressful as they were not confronted with the memories related to being at the hospital as a patient. Ihde in his study [18] explains that when technologies mediate between humans and structures, it also means that technologies play a role in how illness and patient are transformed. This indicates that technologies can transform how patients handle their illness, in our case that the technology can transform the patients to free and active patients as they can stay at home or at their local hospital for consultations. It gave the patients a sense of freedom to do what made sense for them.

### Intimacy at a Distance

One of the most common fears of technology is that machines will replace human contact, making care *cold* by reducing it to mechanical interactions with machines [29]. However, the results of our study show that the patients experience intimacy at a distance, for instance, they state that the doctor was looking intensely at them and that they feel secure that the doctor can see them as the technology allows the doctor to see specific details such as skin changes. This was also found by Pols in a study, where the patients reported that the telecare system had the ability to bring people together and that it functioned as a new communication line to the users [29].

The common etiquette for social interaction such as a handshake is not possible when the consultation is long distance. However, it was not an issue for the patients as they, instead of shaking hands, would wave or use enhanced facial expressions. Having the contact mediated through a screen invited the participants to use other gestures. Ihde explains that technology is shaping our experiences of a situation, and using technology invites humans to act in certain ways [18]. In our study, the technology invited the participants to greet each other in a different way than when meeting face to face. This compensated for the physical contact. Sorknæs [30] also found this when investigating how patients with chronic obstructive lung disease experienced the use of video consultations with nurses.

### Technology

The patients were happy that the health care professionals were in charge of the technology, as they expressed that it could have been a potential stress factor for them. However, as Ihde states [15], handling a new technology is a learning process. Handling a new technology is experienced as stressful until the user has learned how to use it, and the technology using Ihde’s word is *embodied* —meaning that the technology is integrated as a useful tool for the user, for instance, like the remote control for most people.

Another thing to consider as to why the patients were hesitant to being responsible for the technology could have been the fact that it was an unfamiliar device and not a well-known consumer device. Most people use consumer devices in the form of tablets and mobile phones as part of our everyday lives. According to Statistics Denmark [31], the National Statistics Bureau, 4 out of 5 people aged 16 to 89 years used a mobile phone to access the internet in 2016 [32]. What we learned from our results was that the patients did not experience the technology as transparent [18], which is why they were happy that someone else was in charge of the technology. On the other hand, they did not gain experience with handling the technology because the health care professional took responsibility. Therefore, it can be difficult to conclude whether the technology would have been easy to handle for the patients after a learning process.

The majority of the patients wanted to keep the hospital out of their homes, which is why they appreciated the possibility to go to the hospital for the video consultation. The integration process for the patients has different barriers as they experience the technology as a symbol of their illness and not just as a technology. Maybe if the video consultation were accessible on a consumer device, which the participants were used to handling in their everyday lives, it would have been different. However, other studies also point at the fact that patients may have mixed emotions about receiving treatment at home [33,34]. It can also be explained with Heidegger’s explanation of the term “being at home.” Being at home is almost sacred because this is where we experience that we belong [35]. However, some of the patients also stressed that it was a safe place to have conversations with the doctor because this was where they felt at home.

### Strengths and Limitations

The limitation of our study is that it was a small-scale study; however, most qualitative studies are typically small scale. Therefore, despite the small sample size, the aim of this study, as other qualitative studies, was to provide in-depth exploration of the phenomenon under investigation. Therefore, the intention of this study was to understand and explain how patients with hematological diseases experience video consultations.

A selected group of patients who were in a stable period and therefore not representative of all patients with hematological diseases were included, which is why the results can be difficult to generalize to all patients with hematological diseases. Another limitation to the study is that all of the patients in this study had to invest significant time (6 to 8 hours) to attend a face-to-face consultation and that some of these results may not apply to patients with significantly less of a geographic barrier for face-to-face consultations.

However, we have provided rich descriptions of both the context of the study as well as the patients’ experiences with teleconsultations. This lets the readers judge whether the work is possibly transferable to their own settings. The results cannot claim statistical generalizability, but analytical generalization, which emerges by means of the dialectic between theory and practice.

The 2 authors conducted the analysis together to increase the reliability. We presented the analysis process in a table to ensure transparency of the analysis. Quotations from the data were used to link to the participants’ original statements to enhance validity.

### Conclusions and Implications for Practice

There was a lack of evidence on how hematological patients experience video consultations. This new knowledge will benefit both patients and health care professionals in allowing the health care system to provide a more tailored treatment and that will also mean improved flexibility for the patients.

We have gained knowledge of what is important for the patients in terms of seeing the health care professionals through a screen. We found that intimacy can be mediated through a screen, and things other than physical presence matter to the patients. Furthermore, this practice factored how important it is for the patients to have a choice of their own—to be involved in the planning of their own course of treatment.

The patients valued the freedom, and they acted as active patients taking responsibility for their own course of treatment. They experienced that the technology gave them the possibility to feel free and active despite their illness.

## Appendix

Multimedia Appendix 1 Sociodemographic data.

## Abbreviations

IT: information technology
