# Supplementary material for: Use of Video Consultations for Patients With Hematological Diseases From a Patient Perspective: Qualitative Study
Source: J Particip Med. 2018 Dec 19;10(4):e11089. doi: 10.2196/11089 (PMC7434074; doi:10.2196/11089)
Supplement: Multimedia Appendix 1 [file jopm_v10i4e11089_app1.pdf]

Table 3: Socio-demographic data

#	Sex	Age	Marital status	IT-skills	Occupation	Diagnosis	Children	Number of video consultations
1	Woman	87	Widow – lives alone	No	Farmer's wife - retired	Multiple Myeloma	2	5
2	Woman	82	Divorced – lives alone	No	Nurse – retired	Chronic myeloproliferative disorders	1	1
3	Woman	72	Widow – lives alone	Yes	Home care assistant at a nursing home – retired. Works as a volunteer	Chronic myeloproliferative disorders	4	3
4	Man	77	Married	Yes	Teacher and author -retired	Chronic myeloproliferative disorders	2	5
5	Woman	72	Married	Yes	Nurse -retired	Chronic Myeloid Leukemia	2	2
6	Woman	55	Single	No	Unskilled chef – early retirement	Chronic myeloproliferative disorders	0	1
7	Man	69	Married	Yes	High school teacher - retired	Chronic myeloproliferative disorders	2	4
8	Man	70	Divorced – lives alone	Yes	Pre-school teacher -retired	Multiple Myeloma	1	1
9	Woman	78	Widow – lives alone	Yes	Farmer's wife - retired	Lymphoma	2	1
10	Woman	74	Married	Yes	Home care assistant - retired	Chronic Lymphocytic Leukemia	2	1
11	Woman	60	Married	Yes	Healthcare – assistant – actively working	Acute Lymphocytic Leukemia	3	1
12	Man	74	Married	Yes	IT engineer - retired	Lymphoma	2	1
